# Case report: Ovarian mucinous tumor with a mural nodule of liposarcoma: a rare case

**DOI:** 10.3389/fonc.2024.1436854

**Published:** 2024-08-16

**Authors:** Jiezhen Li, Haijian Huang, Qiang Zeng, Xin Chen, Lingfeng Chen

**Affiliations:** ^1^ Provincial Clinical Medical College of Fujian Medical University, Department of Pathology, Fujian Provincial Hospital, Fuzhou, China; ^2^ First Affiliated Hospital of Fujian Medical University, Department of Pathology, Fuzhou, China

**Keywords:** ovarian mucinous tumor, mural nodule, liposarcoma, pathological feature, high-throughput sequencing

## Abstract

**Background:**

Ovarian mucinous tumor with a mural nodule is a rare and special type of ovarian surface epithelial–stromal tumor. Mural nodules are morphologically classified into three types: sarcoma-like, anaplastic carcinomatous, and true sarcomatous nodules. Ovarian mucinous tumors with true sarcomatous mural nodules are rare and challenging to diagnose, with only 10 cases reported worldwide. Currently, liposarcoma mural nodules remain unreported.

**Case presentation:**

A 91-year-old woman was hospitalized for postmenopausal vaginal bleeding for 3 weeks. Imaging revealed a large cystic mass (20.0 cm × 17.7 cm × 12.8 cm) on the right ovary. The mass was multilocular cystic, with a mural nodule (1.4 cm × 1.2 cm × 1.0 cm) in the focal cyst wall. Based on histological morphology, immunohistochemical staining, and MDM2/CDK4 fluorescence *in situ* hybridization testing, the diagnosis was ovarian mucinous cystadenoma with a mural nodule of well-differentiated liposarcoma. To the best of our knowledge, this has never been reported before. High-throughput sequencing identified *KRAS* mutations in the ovarian mucinous cystadenoma. However, the liposarcoma mural nodule did not exhibit *KRAS* mutations but displayed copy number amplifications of *CDK4* and *DDR2*, as well as a frameshift mutation in exon 13 of *ASXL1* (p. A627Gfs*8).

**Conclusions:**

This case broadens the morphological spectrum of mural nodules in ovarian mucinous tumors, deepening our knowledge of this rare morphology. Meanwhile, through high-throughput sequencing, we found no overlapping genetic evidence between the liposarcoma mural nodule and associated ovarian mucinous cystadenoma.

## Background

Mural nodules are single- or multiple-boundary masses arising in the cyst wall. Ovarian mucinous tumors with mural nodules are rare, with an incidence rate of 2 to 5 per million (1). Mural nodules are divided into three histological types based on morphology: sarcoma-like, anaplastic carcinomatous, and true sarcomatous nodules (2–5). Sarcoma-like mural nodules are generally considered benign reactive lesions, whereas anaplastic carcinomatous and true sarcomatous nodules are regarded as invasive malignant tumors (5). Currently, reports of ovarian mucinous tumors with mural nodules of leiomyoma are limited (6, 7). Given the significantly different prognoses associated with various mural nodule types, making an accurate pathological diagnosis of mural nodules is crucial. However, mural nodules are often small and attached to the cyst wall, making them difficult to detect on preoperative imaging examinations. Insufficient sampling by pathologists postoperatively can easily lead to missed diagnoses. Therefore, for ovarian mucinous tumors, pathologists must carefully observe gross specimens, perform adequate sampling, and provide precise diagnoses to optimize patient outcomes.

Currently, only 10 cases of ovarian mucinous tumors with true sarcomatous mural nodules have been reported (**Table 1**), including 3 cases of osteosarcoma mural nodules (9, 10), 3 of fibrosarcoma mural nodules (12–14), 2 of undifferentiated sarcoma mural nodules (8, 14), 1 of rhabdomyosarcoma mural nodule (15), and 1 of high-grade sarcoma mural nodule (11). However, no cases of liposarcoma mural nodules have been reported to date.

**Table 1:** Summary of cases of ovarian mucinous cystic tumor with sarcomatous mural nodules.

References	Yang (2019) (8)	Chu (2021) (9)	McFarland (2015) (10)		Desouki (2014) (11)	Rahilly (1994) (12)	Bruijn (1987) (13)	Prat (1979) (14)		Tsujimura (1992) (15)	Present report
**Number of cases**	1	1	2		1	1	1	2		1	1
**Age (years)**	60	65	34	18	25	69	27	61	49	57	91
**Tumor location**	Right	Right	Left	Left	Right	Left	Left	Right	Left	Left	Right
**Tumor size (cm)**	9.4	25	29	11	44	12	17	16	14.5	15	20
**Nodule size (cm)**	1.6–3.8	5	2	–	3–11	–	4	10	7	15	1.4
**Presenting symptoms**	Lower abdominal discomfort and abdominal distension	Abdominal swelling	Persistent vaginal bleeding and abdominal swelling	Abdominal swelling	Abdominal enlargement and slight distension	Lower abdominal pain	Lower abdominal swelling	Abdominal swelling	Abdominal swelling	Lower abdominal tumor	Vaginal bleeding
**Histologic type of primary** **mucinous ovarian tumor**	Borderline cystadenoma with intraepithelial carcinoma	Mucinous cystadenoma	Cystadenocarcinoma	Borderline cystadenoma	Cystadenocarcinoma	Cystadenocarcinoma	Cystadenocarcinoma	Cystadenoma	Cystadenocarcinoma	Cystadenocarcinoma	Cystadenoma
**Histologic type of** **mural nodule**	Pleomorphic undifferentiated sarcoma	Osteosarcoma	Osteosarcoma	Osteosarcoma	High-grade sarcoma	Fibrosarcoma	Fibrosarcoma	Fibrosarcoma	Undifferentiated sarcoma	Rhabdomyosarcoma	Well-differentiated liposarcoma
**FIGO staging**	IC	IC	IA	IC	IA	IIB	IA	IA	IIIA	IA	IA
**Treatment**	BSO + Omen + ChT	UO	TAH + BSO + Omen	UO	UO	TAH + BSO + appen	USO + Omen	TAH + BSO + appen + RT	TAH + BSO + Omen	TAH + BSO + ChT	TAH + BSO
**Follow-up**	NED, 36 months	NED, 5 months	NED, 18 months	NED, 12 months	–	–	–	Died of hepatic metastases, 18 months	Died of renal failure, 1 week	NED, 3 months	NED, 25 months

-, unknown; appen, appendectomy; BSO, bilateral salpingo-oophorectomy; ChT, chemotherapy; FIGO, International Federation of Gynecology and Obstetrics; NED, no evidence of disease; Omen, omentectomy; RT, radiation therapy; TAH, total abdominal hysterectomy; UO, unilateral oophorectomy; USO, unilateral salpingo-oophorectomy.

Herein, we describe the case of a 91-year-old woman diagnosed with ovarian mucinous cystadenoma with a well-differentiated liposarcoma mural nodule, based on histological morphology, immunohistochemical examination, and MDM2/CDK4 fluorescence *in situ* hybridization (FISH) testing. To the best of our knowledge, this study is the first to report such a case. We compared our case with other cases of true sarcomatous mural nodules to elaborate on the clinicopathological features of this entity, explore the diagnostic and treatment strategies for this rare tumor, and provide reference and insights for future clinical practice.

## Case presentation

A 91-year-old woman was hospitalized due to postmenopausal vaginal bleeding for 3 weeks. Tumor marker assessment revealed a human epididymal protein 4 level of 123.30 pmol/L, risk of ovarian malignancy algorithm (ROMA) premenopausal value of 41.52%, and ROMA postmenopausal value of 31.88%. The preoperative serum levels of CA-125, CEA, CA19-9, and AFP were normal. Abdominal computed tomography revealed a large cystic mass (approximately 20.0 cm × 17.7 cm × 12.8 cm) in the pelvic cavity, with clear margins and a few septations inside the cyst. This large cyst was closely related to the right adnexa. The patient underwent total hysterectomy and bilateral salpingo-oophorectomy under general anesthesia. During surgery, a large cystic mass was detected in the right adnexal region, with a diameter of approximately 20 cm. The cyst was oval-shaped, with a smooth surface and rich blood supply, containing septations. Additionally, its pedicle was connected to the right ovary. No abnormalities were visually observed in the right fallopian tube, uterus, and left adnexa, and no abnormalities were noted in the stomach, small intestine, ileocecal region, colon, rectum, and greater omentum intraoperatively.

**Figure 1:**
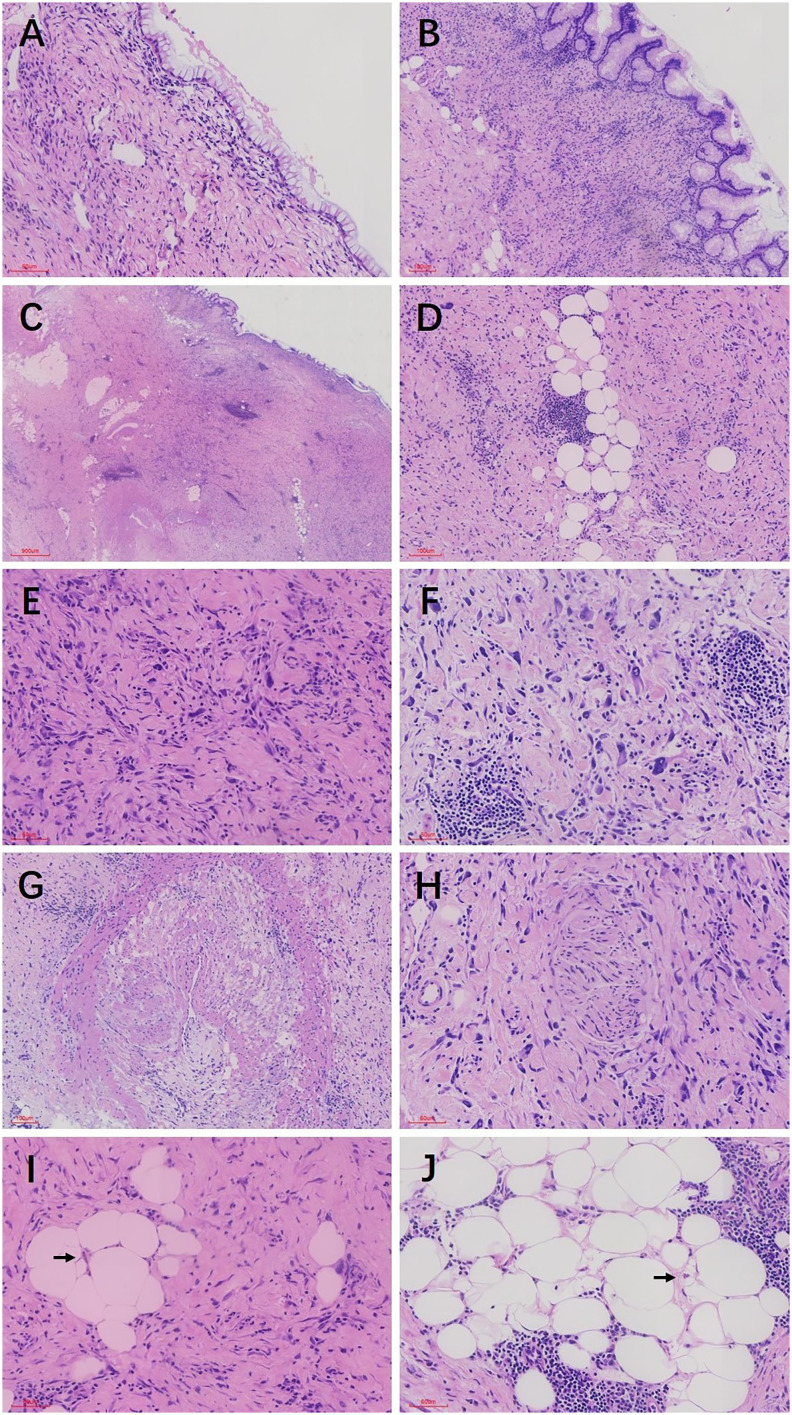
**(A)** The ovarian cyst wall was lined with a single layer of mucinous columnar epithelium, with no significant cellular atypia observed. **(B)** The mucous epithelial cells exhibit a benign morphology, with nuclei located at the basal portion and the cytoplasm rich in mucus **(C)** Mural nodules were present beneath the mucinous epithelium, with unclear boundaries between the nodules and the surrounding tissues. **(D)** The mural nodules consisted of spindle cells, pleomorphic cells, and a few adipocytes. The adipocytes showed good differentiation and varied in size. **(E)** Under high magnification, the spindle cell nuclei exhibited significant atypia, with abundant and eosinophilic cytoplasm, and the tumor stroma demonstrated prominent hyaline degeneration. **(F)** Scattered giant tumor cells were visible. Lymphocyte and plasma cell infiltration and lymphoid follicle formation were noted in the stroma. **(G)** Tumor cells invaded large blood vessels. **(H)** Tumor cells grew around nerves. **(I)** Under high magnification, fat cell nuclei showed mild atypia, with a few visible lipoblasts. **(J)** Adipocytes showed good differentiation and varied in size. Localized nuclei exhibited mild atypia, and lipoblasts were visible. The black arrows in both **I**, **J** indicate lipoblasts.

**Figure 2:**
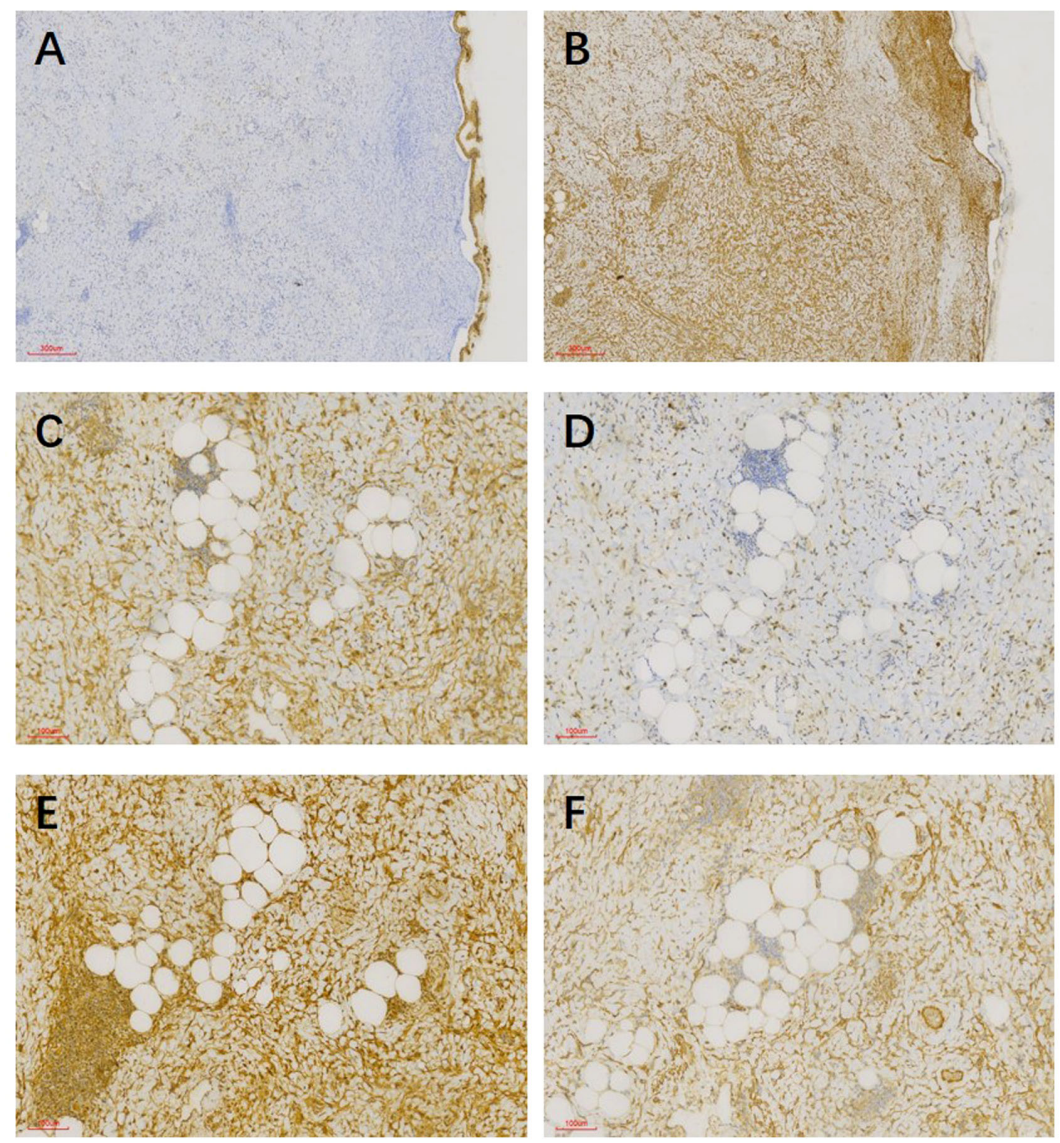
**(A)** Mucinous epithelium expressed cytokeratin, whereas tumor cells in mural nodules did not. **(B)** Mural nodules showed a diffuse strong positive expression of vimentin, whereas mucinous epithelium did not. **(C)** Spindle cells and adipocytes in mural nodules exhibited a strong positive expression of CDK4. **(D)** Spindle cells and adipocytes in mural nodules expressed MDM2. **(E)** Spindle cells and adipocytes in mural nodules exhibited a strong positive expression of P16. **(F)** Spindle cells in mural nodules expressed SMA.

**Figure 3:**
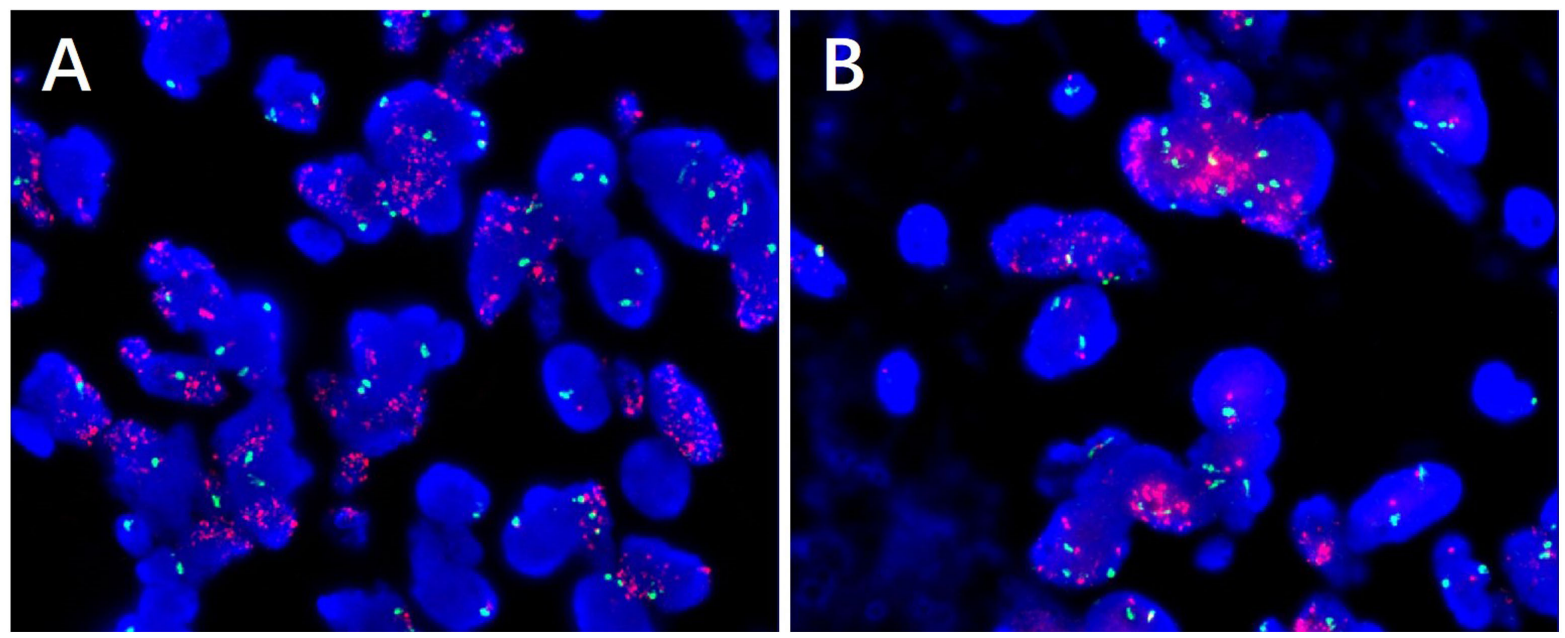
FISH testing revealed *CDK4* **(A)** and *MDM2* **(B)** amplification in mural nodules.

In pathological examination, we first conducted gross examination. We observed a smooth-surfaced, yellowish-brown cystic mass in the right ovary, measuring 20.0 cm × 17.7 cm × 12.8 cm. This mass was composed of multilocular cysts filled with viscous yellowish-brown fluid or transparent mucinous material. Upon careful dissection, we found a mural nodule within the cyst wall in a local region, measuring 1.4 cm × 1.2 cm × 1.0 cm and protruding into the cyst cavity with a clear boundary. The mural nodule had a solid, grayish-white cut surface with a moderate texture. The remaining cyst wall was smooth, without papillary protrusions, and its septum thickness was uneven, ranging from 0.2 to 0.4 cm. Next, (2) microscopic examination was conducted. The ovarian cyst wall was found to be lined with a single layer of gastrointestinal-type mucinous columnar epithelium (**Figure 1A**), showing no papillary hyperplasia. The mucinous epithelial cells exhibited no atypia, with nuclei located at the basal portion and the cytoplasm rich in mucus (**Figure 1B**). A mural nodule was observed beneath the mucinous epithelium. This nodule was composed of short spindle cells, pleomorphic cells, and a few adipocytes (**Figure 1C**). Under low magnification, the spindle tumor cells were indistinguishable from the surrounding tissue and were arranged in sheets or bundles (**Figure 1C**). A few adipocytes were scattered between the spindle cells; they showed good differentiation and varied in size (**Figure 1D**). Under high magnification, the spindle cell nuclei clearly exhibited atypia, with abundant and eosinophilic cytoplasms (**Figure 1E**). Scattered tumor giant cells were also observed (**Figure 1F**). Furthermore, some regions demonstrated signs of tumor invasive growth, including large blood vessel invasion (**Figure 1G**) and perineural growth (**Figure 1H**). The nuclei of adipocytes showed mild atypia under high magnification, with a few visible lipoblasts (**Figures 1I, J**). No definite neoplastic necrosis was observed, and the mitotic figures were approximately 3/10 HPF (high-power fields). In the tumor, interstitial collagen markedly degenerated, accompanied by extensive lymphocytic and plasmacytic infiltration; in some regions, lymphoid follicles formation was noted (**Figure 1A**). In immunophenotyping, the mucinous epithelium expressed cytokeratin (**Figure 2A**) but not vimentin (**Figure 2B**). Conversely, the mural nodule tumor cells did not express cytokeratin (**Figure 2A**) but exhibited diffuse strong positive expression of vimentin (**Figure 2B**). Additionally, the tumor cells in the mural nodule showed a strong positive expression of MDM2 (**Figure 2C**), CDK4 (**Figure 2D**), P16 (**Figure 2E**), MUC4, and SMA (**Figure 2F**), with a Ki-67 proliferation index of 30%. Regarding the FISH test, the mural nodule region showed amplification signals of *CDK4* (**Figure 3A**) and *MDM2* (**Figure 3B**) genes. Combining the histological morphology, immunohistochemistry, and MDM2/CDK4 FISH test results, we diagnosed the patient with ovarian mucinous cystadenoma with a mural nodule of well-differentiated liposarcoma, International Federation of Gynecology and Obstetrics (FIGO) stage IA. High-throughput sequencing was performed separately on the two tumor components. The ovarian mucinous cystadenoma demonstrated the p. G12R, c.34G>C mutation in exon 2 of the *KRAS* gene. Meanwhile, no *KRAS* mutation was observed in the mural nodule of liposarcoma, but rather, amplifications in the copy numbers of *CDK4* and *DDR2*, as well as a frameshift mutation in exon 13 of *ASXL1* (p. A627Gfs*8), were detected.

Regarding treatment and follow-up, the patient did not receive adjuvant therapy postoperatively and was followed-up regularly (the last follow-up was 25 months after surgery). Gynecological ultrasound and tumor marker assessment did not indicate any evidence of recurrence or metastasis.

## Discussion and conclusions

Ovarian mucinous tumor with true sarcomatous mural nodules is an extremely rare tumor entity, with only 10 cases reported currently (8–15). Owing to its rarity and significant variability in prognosis, both diagnosis and treatment are highly challenging. This case report describes in detail the clinicopathological features and diagnostic and therapeutic process of ovarian mucinous cystadenoma with a liposarcoma mural nodule. We aim to explore the pathological diagnosis and treatment strategies for this tumor type and improve the understanding of clinicians and pathologists regarding this tumor.

**Table 1** summarizes cases of ovarian mucinous tumors with true sarcomatous mural nodules (8–15). The onset age range is wide, occurring in women aged 18–91 years (mean age: 51 years). Our patient represents the oldest reported case. Initial clinical manifestations are diverse, primarily presenting as lower abdominal pain or discomfort. In the present case, the clinical presentation was postmenopausal vaginal bleeding. Mucinous ovarian tumors often have a large diameter, ranging from 9.4 to 44.0 cm (mean diameter: 19.4 cm). The mural nodule diameter ranges from 1.4 to 15 cm. The incidence ratio between the left and right ovaries is 6:5. In our patient, the tumor emerged in the right ovary, with a mural nodule diameter of only 1.4 cm, the smallest among all cases. If macroscopic examination is not performed carefully and sampling is inadequate, misdiagnosis is highly likely. According to the FIGO staging system, 6 cases were classified as stage IA, 3 as stage IC, 1 as stage IIB, and 1 as stage IIIA.

Mural nodules can occur in any mucinous ovarian tumors (benign, borderline, or malignant), typically classified into sarcoma-like, anaplastic carcinomatous, and true sarcomatous nodules (2–5). These three mural nodule types are morphologically indistinguishable. The most common are sarcoma-like nodules, which are often multiple and well demarcated from the surrounding mucinous tumor; they generally protrude into the lumen of the mucinous cyst. Microscopically, they are composed of epithelial-like or spindle cells, often mixed with osteoclast-like or ameloblastic giant cells and inflammatory cells (5). Moreover, anaplastic carcinomatous nodules are often poorly defined, large, and infiltrative lesions, with or without lymphovascular invasion. They are usually composed of polygonal cells with high-grade nuclear features; however, the inflammatory infiltration is less prominent (16–18). True sarcomatous mural nodules have wide-ranging morphological spectra. In osteosarcoma mural nodules, atypical spindle-shaped or epithelioid osteoblastic cells can be observed, accompanied with abundant osteoid matrix formation, even lace-like osteoid matrix (9, 10).

Furthermore, fibrosarcoma mural nodules exhibit pleomorphic spindle cells arranged in bundles or fishbone patterns, concurrent with extensive tumor necrosis and mitotic images (12–14). Rhabdomyosarcoma mural nodules demonstrate the typical morphology of rhabdomyoblasts as well as pleomorphic tumor cells, which possess abundant eosinophilic cytoplasm (15).

In the current case, spindle and pleomorphic cells were observed beneath the mucinous epithelium, exhibiting a sheet-like infiltrative growth pattern. Interspersed among the spindle cells are small clusters of adipocytes. Without careful examination, misdiagnosis or overlooking the possibility of liposarcoma is highly possible. Under high magnification, the adipocytes were well differentiated, varying in size, with lipoblasts noted in focal areas. Immunohistochemical staining revealed diffuse strong positivity for CDK4, MDM2, and P16, supporting liposarcoma diagnosis. Further FISH analysis revealed *MDM2* and *CDK4* amplification, thereby confirming the diagnosis of mural nodules in highly differentiated liposarcoma.

Currently, the pathogenesis of ovarian mucinous tumors with mural nodules remains unclear. Mesbah et al. (16) found clonal evidence, including frequent mutations in *KRAS, IDH1, TP53, PTEN*, and *PIK3CA*, in anaplastic carcinomatous nodules and the corresponding ovarian mucinous tumors. Recently, a large-scale series study reported by Chapel et al. (5) showed clonal genetic evidence between various mural nodules and related ovarian mucinous tumors. They suggested no clear and reproducible genetic differences between sarcoma-like, anaplastic carcinomatous, and true sarcomatous mural nodules defined by conventional morphology. Different mural nodule types represent a continuous growth spectrum of ovarian mucinous tumors rather than merely three biologically discrete entities. However, none of the 13 mural nodules reported by Chapel et al. was purely sarcomatous. True sarcomatous mural nodules representing as a dedifferentiated component or a collision of two tumors remains poorly investigated. Desouki et al. (11) reported a case of ovarian mucinous carcinoma with high-grade sarcomatous mural nodules. The mucinous tumor components showed the same *KRAS* mutation as the sarcomatous tumor components, and the sarcomatous mural nodules may represent a form of mucinous tumor dedifferentiation. Whether similar genetic alterations occur when mural nodules exhibit heterologous differentiation (osteosarcoma, rhabdomyosarcoma, and liposarcoma) remains unknown. Currently, the 3 reported cases of osteosarcoma mural nodules (9, 10) and 1 case of rhabdomyosarcoma mural nodule (15) have not undergone relevant molecular testing. Through separate high-throughput sequencing of two distinct tumor components, we found no overlapping genetic evidence between the liposarcoma mural nodule and associated ovarian mucinous cystadenoma. Our study suggests that when the mural nodule of a true sarcoma exhibits heterologous differentiation, it likely represents a collision of two different tumors rather than a form of dedifferentiation of the mucinous tumor.

Considering that ovarian mucinous tumors with true sarcomatous mural nodules are extremely rare, its clinical understanding and comprehension remain insufficient, and unified standards for its treatment strategies remain unavailable. According to the 10 case reports, the primary treatment for ovarian mucinous tumors with true sarcomatous mural nodules is surgical resection (8–15). The surgical approach depends on the tumor stage, extent of involvement, and the need to preserve fertility, with some patients receiving radiotherapy or chemotherapy postoperatively (8, 14, 15). Among the cases of true sarcomatous mural nodules with follow-up records, only two patients died, as reported by Prat et al. (14). One had a fibrosarcoma mural nodule and died of liver metastasis, and the other had an undifferentiated sarcoma mural nodule and died of renal failure. Provenza et al. (19) analyzed the prognosis of 21 cases of ovarian mucinous tumors with anaplastic carcinomatous mural nodules and found that the only significant factor affecting patient prognosis was FIGO staging; the size and histological type of mural nodules, as well as the type of mucinous component and vascular invasion, did not statistically correlate with prognosis. In the present case, adjuvant therapy was not provided after surgical resection, and regular follow-up for 25 months has not revealed evidence of recurrence or metastasis.

In summary, we have reported a rare case of ovarian mucinous tumor with liposarcoma mural nodule, expanding the morphological spectrum of mural nodules. Additionally, through high-throughput sequencing, we found no overlapping genetic evidence between the liposarcoma mural nodule and associated ovarian mucinous cystadenoma.

## Data Availability

The original contributions presented in the study are included in the article/supplementary material. Further inquiries can be directed to the corresponding author.
